# Optimal Trajectory Planning for Wheeled Mobile Robots under Localization Uncertainty and Energy Efficiency Constraints

**DOI:** 10.3390/s21020335

**Published:** 2021-01-06

**Authors:** Xiaolong Zhang, Yu Huang, Youmin Rong, Gen Li, Hui Wang, Chao Liu

**Affiliations:** 1 School of Mechanical Science and Engineering, Huazhong University of Science and Technology, Wuhan 430074, China; xiaolong172@hust.edu.cn (X.Z.); D201677192@hust.edu.cn (Y.H.); D201980282@hust.edu.cn (Y.R.); hustMrwang@hust.edu.cn (H.W.); 2 Guangzhou Institute of Advanced Technology, Chinese Academy of Sciences, Guangzhou 511400, China; gen.li@giat.ac.cn

**Keywords:** trajectory planning, localization uncertainty, energy efficiency, dolphin swarm optimization, swarm intelligence, wheeled mobile robot

## Abstract

With the rapid development of robotics, wheeled mobile robots are widely used in smart factories to perform navigation tasks. In this paper, an optimal trajectory planning method based on an improved dolphin swarm algorithm is proposed to balance localization uncertainty and energy efficiency, such that a minimum total cost trajectory is obtained for wheeled mobile robots. Since environmental information has different effects on the robot localization process at different positions, a novel localizability measure method based on the likelihood function is presented to explicitly quantify the localization ability of the robot over a prior map. To generate the robot trajectory, we incorporate localizability and energy efficiency criteria into the parameterized trajectory as the cost function. In terms of trajectory optimization issues, an improved dolphin swarm algorithm is then proposed to generate better localization performance and more energy efficiency trajectories. It utilizes the proposed adaptive step strategy and learning strategy to minimize the cost function during the robot motions. Simulations are carried out in various autonomous navigation scenarios to validate the efficiency of the proposed trajectory planning method. Experiments are performed on the prototype “Forbot” four-wheel independently driven-steered mobile robot; the results demonstrate that the proposed method effectively improves energy efficiency while reducing localization errors along the generated trajectory.

## 1. Introduction

In the intelligent manufacturing field, wheeled mobile robots are one of the most widely used groups of robots in factories and warehouses to perform material transportation tasks that benefit from their automation and efficiency [1]. As one of the key technologies of mobile robots, trajectory planning has recently attracted plenty of research. A series of trajectory planning schemes have been reported until now, such as the graph search-based method [2,3], interpolating curve planning method [4,5], sampling-based planning method [6], and numerical optimization method [7]. Among these methods, the interpolating curve planning method is a widely examined planning strategy due to its optimized performance and strong ability to handle external constraints. In [8], the authors propose a spline-based trajectory planning method to guarantee constraint satisfaction for autonomous guided vehicles. In [9], a minimum time trajectory planning method is proposed for a two-wheeled mobile robot by combining a straight line and a Bézier curve under the torque constraints. Although the interpolating curve planning method offers potential ways for trajectory generation, the trajectory optimization brings a heavy calculation burden owing to the nonlinearity, non-differentiability, and multi-objective requirements of the wheeled mobile robot system [10]. Swarm intelligence algorithms are proved to be a solution for this challenge [11]. In [12], the authors propose a novel chaotic grouping particle swarm optimization algorithm with a dynamic regrouping strategy to solve complex numerical optimization problems. In [13], a new evolutionary computation approach based on an artificial bee colony algorithm for solving the multi-objective orienteering problem is presented. In [14], the authors first propose a dolphin swarm algorithm (DSA) based on the biological characteristics and living habits of dolphins to solve the optimization problem with first-slow-then-fast convergence. The DSA simulates the predatory process of dolphins for multi-dimensional numerical optimization problems with four phases, i.e., search, call, reception, and predation. Many practical engineering optimization problems can be solved by DSA due to its high efficiency, low computational burden and excellent optimization performance [15,16]. In [16], the authors optimize the localization process by using DSA to process the sensed data in Wireless Sensor Networks. However, in the process of dolphin updating, the positions of the dolphins are updated based on a random method. Thus, the efficiency and convergence of the DSA decrease and the obtained solution may lead to low optimization accuracy or even failure [17]. Moreover, dolphins are explored completely based on fixed step size during the searching stage; this results in an inability to perform the searching efficiently.

Reliable localization is a fundamental requirement for mobile robots during autonomous navigation. Large localization errors or localization failure will lead to excessive tracking errors of mobile robots and even safety accidents. Nevertheless, most existing localization methods are flawed in terms of applicability to ambiguous environments, such as long corridors and empty areas [18]. This may result in localization uncertainty for mobile robots. The localization ability of the mobile robot, i.e., the localizability of the robot, is used to measure localization uncertainty in the environment. It varies over a given map since environmental information has different effects on robot localization at different positions [18,19]. In ambiguous environments, it is of importance to take localizability into consideration to avoid large localization errors on the planned trajectory. The early effort of this is presented in [20], where the authors first provide theoretical analysis for localization accuracy based on Cramér–Rao Bound. An ambiguity model of the indistinguishability is presented to formalize and quantify the perceptual ambiguity in the static environment [21]. In [22], this paper proposes a novel method for real-time localizability estimation by analyzing the constraints in each direction to predict localization performance. In [23], a minimum uncertainty planning method is proposed based on a partially observable Markov decision process (POMDP) with continuous observation spaces. In [24], the authors present an uncertainty-augmented Markov Decision Process to approximate POMDP to estimate the localization of the robot. However, POMDPs may not be computationally achievable owing to the expansion of the state space, although they are theoretically satisfied.

Energy efficiency is a crucial technology for wheeled mobile robots to run automatically for a long period. It is significant to optimize energy consumption due to the limited capacity of the equipped batteries. To extend the running time of the robot system, researchers have made many contributions to the energy efficiency of wheeled mobile robots. An energy optimal trajectory generation method is presented for a two-wheel differential mobile robot by constructing a cost function related to the integral of the Lagrangian [25]. As reported in [26], the authors propose a minimum-energy translational and rotational velocity trajectory planning for three-wheeled omnidirectional mobile robots by using Pontryagin’s minimum principle. In [27], a closed-form trajectory planning method based on Dubins’ path is presented to obtain the minimum energy trajectory for car-like robots. However, in the research studies mentioned above, very little attention has been devoted to building a generic energy model of different styles of wheeled mobile robots to provide a solid foundation for energy efficiency efforts.

Localizability and energy efficiency may be conflicting trajectories for optimization goals. Energy efficiency is mainly affected by speed, ground friction conditions, etc [28]. However, the localizability of the robot is chiefly influenced by the environmental map information. As a matter of fact, meeting energy efficiency may reduce the reliability of localization along the trajectory and vice versa. Therefore, how to balance energy efficiency and localizability poses a crucial challenge to trajectory planning, which motivates us to make a contribution in this paper.

The aim of this paper is to explore an effective trajectory planning method, considering energy efficiency and localizability simultaneously to bring the wheeled mobile robot from the start position to the goal position. The main contributions of this paper can be reflected in: (1) A novel localizability measure method is proposed based on the likelihood function to explicitly quantify the influence resulting from environmental information on the localization of mobile robots. (2) By utilizing the localizability measure method, the localizability aware map (LAM) is achieved such that the localization error at a given position can be effectively estimated. (3) An optimal trajectory planning method is proposed under localization uncertainty and energy efficiency constraints by incorporating an improved dolphin swarm algorithm (DSA) into the trajectory optimization process. The DSA utilizes the proposed adaptive step strategy and learning strategy to minimize the cost function during the robot motions. (4) Implemented on a developed prototype “Forbot” mobile robot, comprehensive experiments demonstrate that the proposed trajectory planning method guarantees the minimum total cost to balance localization uncertainty and energy efficiency while the robot navigates along the trajectory.

The rest of this paper is organized as follows. Section 2 explains how to establish the energy consumption model and describes the localizability measure method. In Section 3, the path planning with the energy and localizability criteria is developed. The proposed trajectory optimization based on the improved DSA is explained in detail in Section 4. Experimental results of the proposed trajectory planning method are shown and performances of the proposed method are analyzed in Section 5. Concluding remarks and future work are given in Section 6.

## 2. Energy Model and Localizability Measure

### 2.1. Energy Model

**Figure 1:**
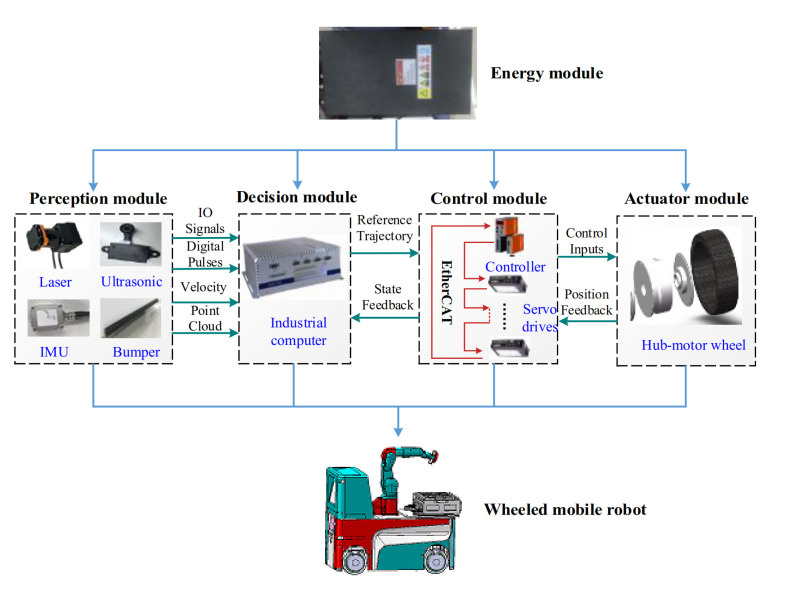
Hardware architecture.

As shown in Figure 1, the common hardware architecture for mobile robots mainly consists of a perception module, decision module, control module, actuator module, and energy module. The energy module, equipped with batteries, and the battery management system provide electrical energy to other modules to drive the robot. The total energy consumption $E_{t}(t)$ of the wheeled mobile robot is derived as [28]:

(1) $$E_{t}(t)=E_{k}(t)+E_{a}(t)+E_{e}(t),$$

where $E_{k}$, $E_{a}(t)$ and $E_{e}(t)$ are kinematic energy consumption, actuators’ energy consumption and nonmechanical devices’ energy consumption, respectively, which are defined as follows:

(2) $$\left\{\begin{array}{l} E_{k}(t)=\int \left(m\max\left(v(t)a(t),0\right)+I\max\left(\gamma (t)\delta (t),0\right)\right)dt\\ E_{a}(t)=\int \left(\sum_{i=0}^{N}\mu \frac{mg}{N}v_{w}{}_{i}\right)dt\\ E_{e}(t)=P_{e}t \end{array}\right.,$$

where v and γ denote the x-direction and yaw velocities, respectively. m and I represent the mass and the rotational inertia of the mobile robot, separately. a and δ are the x-direction and yaw accelerations, respectively. μ denotes the coefficient of the rolling friction influenced by the ground types. $v_{w}{}_{i}$ denotes the linear velocity of the ith wheel. N is the number of wheels. g is the gravitational acceleration. $P_{e}$ defines the total power of nonmechanical devices.

### 2.2. Localizability Measure

In the field of robot navigation, mobile robots may inevitably run in ambiguous environments that include symmetrical or featureless map regions, resulting in the perceptual aliasing of external sensors. Therefore, the localization errors may accumulate in such regions, which brings a high risk of choosing a path without considering localization uncertainty [18]. Given that the perceptual aliasing region causes relatively strong localization confusion, it is necessary to avoid such regions as much as possible to ensure safety when the robot runs automatically on the planned path. Then, the probability of localization confusion (LCP) is proposed to estimate the probability of the specific event A, i.e., a robot’s true pose is confused with some other poses over a grid map based on a given observation model. To be more mathematically precise, we provide the following definitions.

**Definition** **1.** *Let* $z_{x_{a}}$ *be the observation data captured by the robot-mounted sensors at the pose* $x_{a}$ *. Considering an input pose* $x_{m}$ *as the independent variable, the likelihood function for sensor-based localization can be defined as [21]*

(3) $$L(x_{m})≜P(z_{x_{a}}|x_{m}).$$

According to (3), we can infer that if the arbitrary value of $L(x_{m})$ for a given $x_{a}$ is smaller than $L(x_{a})$, the pose of the robot can be accurately obtained via the commonly used maximum likelihood estimation method. Unfortunately, $L(x_{m})\geq L(x_{a})$ may occur as a result of the mentioned ambiguous environments such that the maximum likelihood estimation method may confuse the pose $x_{m}$ with the actual pose $x_{a}$.

**Definition** **2.** *Assume that*ε*is a small scalar used to compensate for the unmodeled factors; for the event*$A:\;L(x_{m})+\varepsilon \geq L(x_{a})$*, we define the LCP as follows*

(4) $$P_{x_{m}|x_{a}}^{A}≜P(L(x_{m})+\varepsilon \geq L(x_{a})),$$

*where*$x_{a}$*and*$x_{m}$*are deterministic values, and the only random variable is*$z_{x_{a}}$*because of sensor measurement uncertainty. If the observation data*$z_{x_{a}}$*is known, the likelihood value of*$L(x_{m})$*can be uniquely calculated. So, we can conclude that the LCP is dependent on the distribution of*$z_{x_{a}}$.

Then, the LCP defined by (4) can be rewritten based on Monte Carlo integration as follows:

(5) $$P_{x_{m}|x_{a}}^{A}\approx \frac{1}{N_{p}}\sum_{i=1}^{N_{p}}I_{A}(z_{x_{a}}^{i}),$$

where $z_{x_{a}}^{i}$ represents the ith sample of the observation data. $N_{p}$ is the number of all sampling.$I_{A}(z_{x_{a}})$ denotes an indicator function, as depicted below [24]:

(6) $$I_{A}(z_{x_{a}})=\left\{\begin{array}{l} 0\;\;L(x_{m})+\varepsilon <L(x_{a})|z_{x_{a}}\\ 1\;\;L(x_{m})+\varepsilon \geq L(x_{a})|z_{x_{a}} \end{array}\right..$$

**Figure 2:**
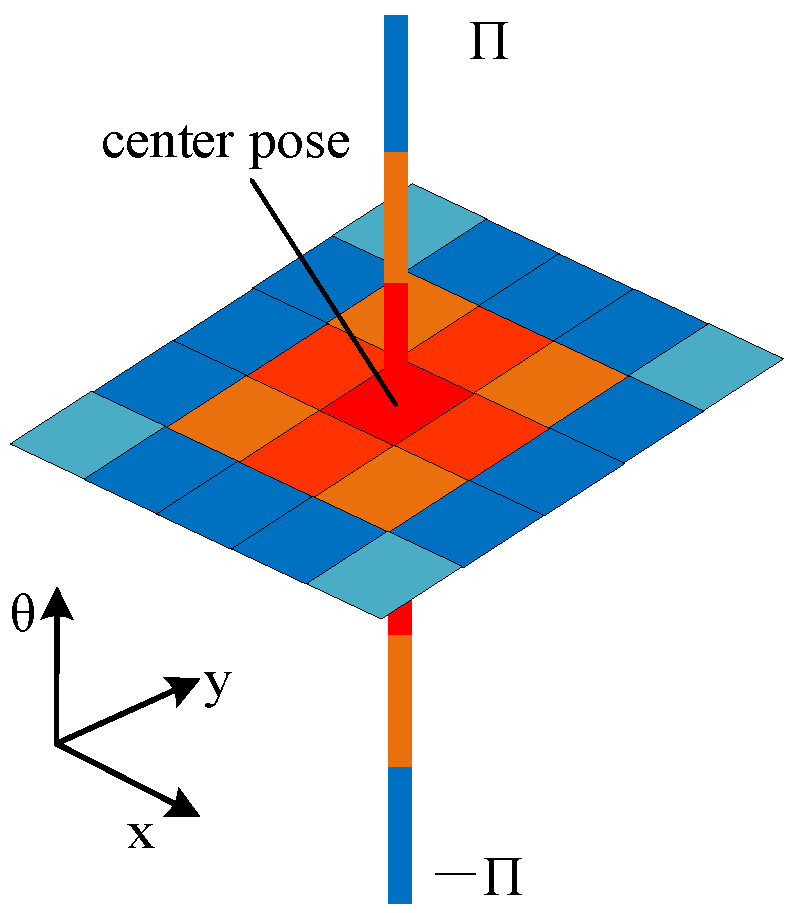
Schematic diagram of the set Ω.

To quantitatively estimate the localization quality of poses over the map, we adopt LCP as the weight to compute the localizability evaluation value (LEV) as follows:

(7) $$\mathrm{LEV}(x)=\frac{\sum_{\Delta x_{i}\in \Omega }\Gamma (x+\Delta x_{i})P_{x+\Delta x_{i}|x}^{A}}{\sum_{\Delta x_{i}\in \Omega }P_{x+\Delta x_{i}|x}^{A}},$$

where $\Delta x_{i}≜(\Delta x_{i},\Delta y_{i},\Delta \theta_{i})$ is the ith incremental pose relative to x. The difference between x and $x+\Delta x_{i}$ determined by Γ(x+Δxi)=(Δxi2+Δyi2)12+δ|Δθi| with a coefficient δ used to transform the orientation difference into the distance difference. As shown in Figure 2, the center of the diagram represents the actual pose and we can find that the closer the color is to red, the smaller the Γ value at that pose. Ω denotes a set including all $\Delta x_{i}$ as follows:

(8) Ω=Δxi|((Δxi2+Δyi2)12≤Φd(Ω)&Δθ=0)∩(|Δθi|≤Φθ(Ω)&(Δxi2+Δyi2)12=0)

where $\Phi_{d}(\Omega )$ and $\Phi_{\theta }(\Omega )$ are the distance range and the orientation range of the set Ω, respectively.

LEV(x) is a criterion that reflects the expected localization performance based on the given observation model at the pose x. According to (7), we can conclude that the LEV(x) represents the weighted average localization error resulting from the ambiguity between the actual pose x and the other poses from Ω. A larger LEV(x) implies a larger localization error caused by perceptual aliasing when a robot passes the pose x.

### 2.3. The Localizability-Aware Map Construction

The LEV is achieved by the aforementioned localizability evaluation method so that the localization reliability can be evaluated, and the unambiguous areas over the occupancy grid map (OGM) can be identified. However, the calculated values of the LEV cannot be used directly for path planning, due to the large fluctuations of the LEV. To derive LAM, the process is explained in detail as listed below:

(1) *OGM Building:* The generation of the OGM is an increasingly mature technology in the field of robotics. In this paper, the simultaneous localization and mapping method presented in [29] is adopted to build the OGM of the environment.

(2) *LEV Calculation:* To calculate the LEV over the generated OGM by Equation (7), the specific calculation method of $P(z_{x_{a}})$ needs to be determined.

As investigated in [30], sampling large amounts of data is time-consuming for a 2-D LIDAR in a large-scale environment and it is noted that the beam model complies with the physical measurement model of LIDAR. In this research, the beam model is adopted as the observation model to perform the calculation of $P(z_{x_{a}})$. Then, we consider the parameters ε, $\Phi_{d}(\Omega )$ and $\Phi_{\theta }(\Omega )$. The δ specifies as $\delta =\tau_{d}/\tau_{o}$ where $\tau_{d}$ and $\tau_{o}$ are the maximum tolerable distance and orientation errors, respectively. Since LEV(x) is the weighted average localization error, it can be inferred that inequality $\mathrm{LEV}(x)\leq \Phi_{d}(\Omega )+\delta \Phi_{\theta }(\Omega )$. By combining the inequality with (7), we have $\tau_{d}\leq \Phi_{d}(\Omega )$ and $\tau_{o}\leq \Phi_{\theta }(\Omega )$. To improve computing efficiency, we select $\Phi_{d}(\Omega )=\tau_{d}$ and $\Phi_{\theta }(\Omega )=\tau_{o}$. In this paper, the related parameters are set as $N_{p}=20$, ε=0.5, $\Phi_{d}(\Omega )=0.25\;m$, $\Phi_{\theta }(\Omega )=5^{\circ }$. It is noted that we calculate the LEV by integrating over angles, i.e., $\mathrm{LEV}(x)=\int \mathrm{LEV}(x,y,\theta )d_{\theta }$, since this paper does not use angle information.

(3) *Normalization:* To adjust values measured on different scales to a notionally common scale, the LEV is normalized between 0 and 1, representing lower and higher localizability, respectively.

(4) *LEV Smoothing:* If a robot enters the connected regions with fluctuating LEVs, the robot may fall into local oscillations during motion planning. In this step, the Gaussian Filter with a mask is used to smooth the values of LEV to suppress oscillations. After this step, the LAM graph is obtained to provide localizability constraints for path planning.

**Figure 3:**
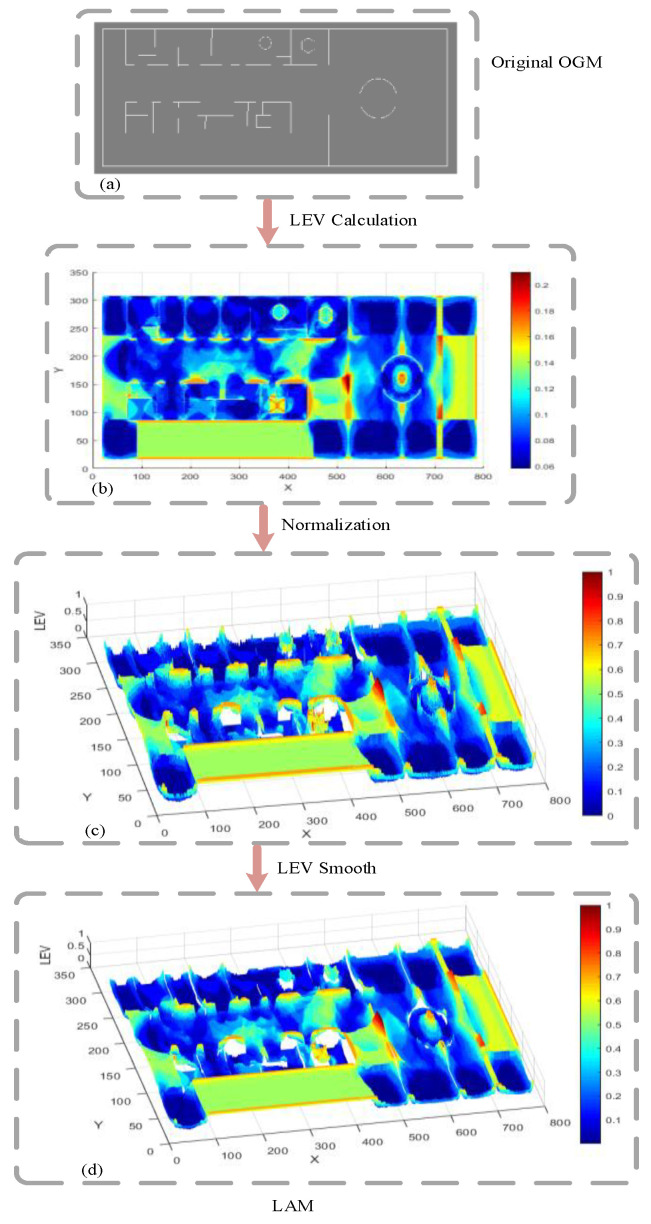
The construction process of the LAM. (**a**) Original OGM; (**b**) LEV calculation map; (**c**) LEV smooth map; (**d**) LAM.

Figure 3 illustrates the construction process of the LAM graph. Figure 3a is the original OGM with 308 × 786 pixels used to represent the environment in the form of block grids; each grid is either occupied or unoccupied. By calculating the LEV, we have Figure 3b that shows the LEV of each grid (ranging from 0.06 to 0.21). Normalization and LEV smoothing are then performed. As shown in Figure 3d, the LEV is getting larger as the color changes from blue to red, that is, the localizability is getting worse.

## 3. Optimal Path with Localization Uncertainty and Energy Efficiency

In what follows, a heuristic search algorithm is examined with the novel localizability and energy related criterion to search for an optimal motion path by employing the aforementioned localizability and energy model constraint.

### 3.1. Heuristic Search Algorithm

A search algorithm that exploits a heuristic function at each node is often called a heuristic search algorithm; it has better computational complexity than brute-force algorithms. The A* algorithm, known to belong to this category of algorithms, has become a ubiquitous searching technology in path planning in the mobile robots field [31]. By searching all possible solutions, the $A^{*}$ algorithm determines a least-cost path based on the specific cost index from the start node to the goal node in a grid map. Based on this property, a novel cost function considering important factors, such as energy consumption and localizability, is designed to obtain an optimal path for mobile robots. 

As a heuristic algorithm, the cost function f(⋅), which is an estimate for the importance of the candidate node in the path, is one of the key elements of the $A^{*}$ algorithm. In this report, we employ a grid map to describe the distribution of the obstacles, in which each grid that is either occupied by obstacles or is free is taken into account as a node n. By offering a search order, the function f(⋅) represents a cost of the path starting from the start node $n_{s}$ to the goal node $n_{g}$, which includes the actual cost g(n) and the heuristic cost h(n), i.e.:

(9) f(n)=g(n)+h(n)

worked out as follows:

(10) $$g(n_{i})=g(n_{i}-1)+s_{n_{i}}$$

where $s_{n_{i}}$ is the travel distance between the node $n_{i}-1$ and the node $n_{i}$.

However, the shortest path may not mean minimum energy consumption or a low risk of failure to perform localization in some scenarios. To avoid large localization errors and large energy consumption, it is essential to consider the factors that have a great influence on the localization process and energy consumption when planning a path. Therefore, a novel cost function considering both localizability and energy efficiency will be presented to plan a path for mobile robots.

### 3.2. Path Planning

To satisfy the demands of considering localizability and energy efficiency simultaneously for the robot path planning, the actual cost model (10) is redesigned as:

(11) $$g(n_{i})=g(n_{i}-1)+\alpha_{1}C_{l}+\alpha_{2}C_{e},$$

where $C_{l}$ and $C_{e}$ denote the localization-related term and energy-related term, respectively. $\alpha_{1}$ and $\alpha_{2}$ are the coefficients to balance the weight between $C_{l}$ and $C_{e}$, which are defined by:

(12) $$C_{l}=\mathrm{LEV}(n_{i}),$$

(13) $$C_{e}(n_{i})=2\eta (n_{i})\mu_{n_{i}-1,n_{i}}mg\int |v_{r}(t)|dt=2\eta (n_{i})\mu_{n_{i}-1,n_{i}}mgs_{n_{i}-1,n_{i}}\;,$$

where $\mu_{n_{i}-1,n_{i}}$ denotes the friction parameter, $s_{n_{i}-1,n_{i}}$ is the length of the path segment between the nodes $n_{i}-1$ and $n_{i}$. $\eta (n_{i})$ is a penalty factor to maintain a safe distance from surrounding obstacles and is expressed as:

(14) $$\eta (n_{i})=\left\{\begin{array}{ll} 1, & d_{o}>D_{s}\\ \frac{D_{s}-D_{m}}{d_{o}-D_{m}}, & D_{m}<d_{o}\leq D_{s}\\ 1000, & d_{o}\leq D_{m} \end{array}\right.,$$

where $d_{o}$ denotes the distance between the current node $n_{i}$ and the nearest obstacle. $D_{s}$ and $D_{m}$ are the maximum and minimum safety distance, respectively. $d_{o}>D_{s}$ indicates that the current node is far away from obstacles. If $D_{m}<d_{o}\leq D_{s}$, the path cost increases with the increase in $d_{o}$. The current node is too close to the obstacle when $d_{o}\leq D_{m}$. 

Next, we focus on the heuristic function $h(n_{i})$. As investigated in [32], the heuristic function is admissible if it does not overestimate the cost of reaching the goal node from a particular node. The ideal situation is the heuristic function evaluating the accurate cost. However, in most of the robot planning problems, it is impractical to find such heuristic functions. Thus, the heuristic effectiveness and computational efficiency of heuristic search algorithms depend on the selection of the heuristics.

A new localizability-energy-related heuristic cost function is proposed as:

(15) $$h(n_{i})=\beta_{1}\mathrm{LEV}(n_{i})+\beta_{2}\mu_{n_{i}-1,n_{i}}mgs_{n_{i},n_{g}},$$

where $\beta_{1}$ and $\beta_{2}$ are the weighted coefficients. $s_{n_{i},n_{g}}$ denotes the distance between node $n_{i}$ and the goal node $n_{g}$.

In summary, we present the novel cost function $f(n_{i})$ by combining (11) and (15) as follows:

(16) $$f(n_{i})=g(n_{i}-1)+\alpha_{1}C_{l}+\alpha_{2}C_{e}+\beta_{1}\mathrm{LEV}(n_{i})+\beta_{2}\mu_{n_{i}-1,n_{i}}mgs_{n_{i},n_{g}}.$$

By using the cost function (16), an optimal collision-free path with localizability awareness and energy efficiency can be achieved.

## 4. Trajectory Optimization with Improved DSA 

### 4.1. Parameterized Trajectory

The path generated by the heuristic search method is often a piecewise linear path or even a sharp path. The mobile robot needs to start, stop, and rotate frequently due to the discontinuity of the path, resulting in time delay, energy consumption, and unnecessary wear on the robot parts. To handle these problems, we employ a parameterized cubic Bézier curve to smooth the path, which benefits from the continuity and local controllability of the Bézier curve. Furthermore, the trajectory is further optimized by explicitly taking into account localizability and energy efficiency.

**Figure 4:**
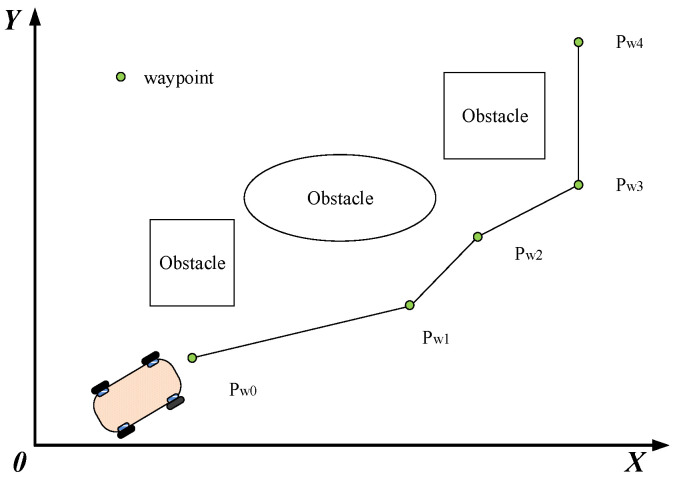
Selection of waypoints.

A series of Bézier curves are inserted between each segment defined by two consecutive waypoints and connected to obtain a complete smooth trajectory. As shown in Figure 4, the knee points along the generated path are chosen as waypoints. If two near waypoints are extremely close, they are merged into one waypoint. On the other hand, if a path segment is too long, new waypoints need to be inserted [33]. Thus, we can achieve a series of generated waypoints expressed by $P_{w0},\cdots ,P_{wi},\cdots ,P_{wN}$. Further, we define the angular bisector of the two near segments as the orientation of the corresponding waypoints.

The cubic Bézier curve passes through the initial waypoint $P_{i-1}^{w}={\left[x_{i-1}^{w},y_{i-1}^{w},\theta_{i-1}^{w}\right]}^{T}$ and finial waypoint $P_{i}^{w}={\left[x_{i}^{w},y_{i}^{w},\theta_{i}^{w}\right]}^{T}$ of a path segment. Its sharpness can be reshaped by adjusting the two control points $P_{i1}^{c}={\left[x_{i1}^{c},y_{i1}^{c}\right]}^{T}$ and $P_{i2}^{c}={\left[x_{i2}^{c},y_{i2}^{c}\right]}^{T}$. [x,y] and θ denote the position and orientation of the corresponding waypoint, respectively. Then, the parameterized trajectory of the cubic Bézier curve is designed as follows:

(17) $$\left\{\begin{array}{c} x(\tau_{i})={\left(1-\tau_{i}\right)}^{3}x_{i-1}^{w}+3\tau_{i}{\left(1-\tau_{i}\right)}^{2}x_{i1}^{c}+3\tau_{i}^{2}(1-\tau_{i})x_{i2}^{c}+\tau_{i}^{3}x_{i}^{w}\\ y(\tau_{i})={\left(1-\tau_{i}\right)}^{3}y_{i-1}^{w}+3\tau_{i}{\left(1-\tau_{i}\right)}^{2}y_{i1}^{c}+3\tau_{i}^{2}(1-\tau_{i})y_{i2}^{c}+\tau_{i}^{3}y_{i}^{w} \end{array}\right..$$

Subject to:

(18) $$\begin{array}{l} \frac{dx(\tau_{i})}{dt}{|}_{t=T_{i-1}}=\frac{3(x_{i1}^{c}-x_{i-1}^{w})}{T_{i}-T_{i-1}}=v_{i-1}\cos\theta_{i-1}^{w}\\ \frac{dy(\tau_{i})}{dt}{|}_{t=T_{i-1}}=\frac{3(y_{i1}^{c}-y_{i-1}^{w})}{T_{i}-T_{i-1}}=v_{i-1}\sin\theta_{i-1}^{w}\\ \frac{dx(\tau_{i})}{dt}{|}_{t=T_{i}}=\frac{3(x_{i}^{w}-x_{i2}^{c})}{T_{i}-T_{i-1}}=v_{i}\cos\theta_{i}^{w}\\ \frac{dy(\tau_{i})}{dt}{|}_{t=T_{i}}=\frac{3(y_{i}^{w}-y_{i2}^{c})}{T_{i}-T_{i-1}}=v_{i}\sin\theta_{i}^{w} \end{array}$$

where $x(\tau_{i})$ and $y(\tau_{i})$ are the trajectory with parameter $\tau_{i}=\frac{t-T_{i-1}}{T_{i}-T_{i-1}}\in [0,1],t\in [T_{i-1},T_{i}]$ in the x and y direction, respectively. $T_{i-1}$ and $T_{i}$ are the arrival time at each waypoint for the segment $P_{i-1}^{w}P_{i}^{w}$. Then, when t varies from $T_{i-1}$ to $T_{i}$, the trajectory varies from $P_{i-1}^{w}$ to $P_{i}^{w}$. $v_{i-1}$ and $v_{i}$ are the velocities at waypoints $P_{i-1}^{w}$ and $P_{i}^{w}$, respectively.

It is noted that the control points $P_{i1}^{c}$ and $P_{i2}^{c}$ are dependent on the arrival time $T_{i}$ and the velocity $v_{i}$. According to (18), we can obtain the control points as follows:

(19) $$\begin{array}{l} x_{i1}^{c}=x_{i-1}^{w}+\frac{1}{3}(T_{i}-T_{i-1})v_{i-1}\cos\theta_{i-1}^{w}\\ y_{i1}^{c}=y_{i-1}^{w}+\frac{1}{3}(T_{i}-T_{i-1})v_{i-1}\sin\theta_{i-1}^{w}\\ x_{i2}^{c}=x_{i}^{w}-\frac{1}{3}(T_{i}-T_{i-1})v_{i}\cos\theta_{i}^{w}\\ y_{i2}^{c}=y_{i}^{w}-\frac{1}{3}(T_{i}-T_{i-1})v_{i}\sin\theta_{i}^{w} \end{array}$$

Consequently, by choosing suitable $T_{i}$ and $v_{i}$, we will achieve an optimized trajectory such that the mobile robot can approach the final goal $P_{wN}$ along all these waypoints.

### 4.2. Improved DSA

In this section, we present a trajectory smoothing method based on improved DSA by selecting parameters $T_{i}$ and $v_{i}$ while minimizing the localization error and minimizing energy consumption. The energy model (1) is calculated by combining the designed Bézier curve (17) as follows:

(20) $$\begin{array}{l} E(T_{i},v_{i})=E_{k}+E_{a}+E_{e}\\ =\int_{0}^{T_{i}}\left(m\max\left(v_{i}(\tau_{i})a(\tau_{i}),0\right)+I\max\left(\gamma (\tau_{i})\delta (\tau_{i}),0\right)\right)dt+\int_{0}^{T_{i}}\sum_{j=0}^{N}\mu \frac{mg}{N}v_{i}(\tau_{i})dt+P_{e}T_{i} \end{array}$$

Then, we aim to solve the following optimization problem:

(21) $$\min J(T_{i},v_{i})=\min(\rho_{1}\int \mathrm{LEV}(p(\tau_{i}))d\tau_{i}+\rho_{2}E(T_{i},v_{i}))$$

where J is objective function. $p(\tau_{i})=(x(\tau_{i}),y(\tau_{i}))$ denotes the point on the parameterized trajectory. $\rho_{1}$ and $\rho_{2}$ are the coefficients to balance the weight between and localizability and energy consumption.

To find the most appropriate $T_{i}$ and $v_{i}$, we present an improved DSA to enhance optimization efficiency. The DSA simulates the predatory process of dolphins for multi-dimensional numerical optimization problems with four phases, i.e., search, call, reception, and predation. This algorithm has numerous advantages of few parameters and strong search capability, which makes it have better global search ability and better stability compared with the conventional evolutionary algorithms 14.

For the DSA, for each dolphin $Dol_{i}(i=1,2\cdots ,N_{dol})$, we define $L_{i}$ as the individual optimal solution that $Dol_{i}$ finds in a single time and define $K_{i}$ as the neighborhood optimal solution. Then, we have three types of distances, i.e., $DD_{i,j}=\left\|Dol_{i}-Dol_{j}\right\|$, $DK_{i}=\left\|Dol_{i}-K_{i}\right\|$ and $DKL_{i}=\left\|L_{i}-K_{i}\right\|$. 

In the search stage, a sound wave mechanism is employed between the individual dolphin $Dol_{i}$ and a new child dolphin $X_{ijt}$. More specifically, the sound is defined as $Vd_{j}$ (j=1, ⋯, M), where M is the number of sounds. A maximum search time $T_{sm}$ is set to prevent the DSA from falling into the search stage. Within the maximum search time $T_{sm}$, the dolphin $Dol_{i}$ will search a new child solution $X_{ijt}$ based on the sound wave during the propagation time t, namely:

(22) $$X_{ijt}=Dol_{i}+Vd_{j}t$$

After calculating the fitness $Fit(X_{ijt})$ of the new solution $X_{ijt}$, we can obtain the optimal fitness as:

(23) $$Fit_{iab}=\min_{j=1,2,\cdots ,M;t=1,2,\cdots ,T_{sm}}Fit(X_{ijt})$$

Then, the individual optimal solution $L_{i}$ is specified as $L_{i}=X_{iab}$. If $Fit(L_{i})<Fit(K_{i})$, the neighborhood optimal solution $K_{i}$ is replaced by $L_{i}$.

In the call stage and reception stage, each dolphin informs other dolphins, through sound wave, as to whether a better solution is found and where it is located. The detailed process can be found in the literature [14].

In the predation stage, each dolphin updates its own location to hunt for preys within a certain surrounding radius $R_{2}$. Besides, the maximum range $R_{1}$ is set as $R_{1}=T_{sm}\times \left\|Vd_{j}\right\|$. The update process is divided into three cases based the distance $DK_{i}$.

(1) If $DK_{i}\leq R_{1}$, a new dolphin $newDol_{i}$ is derived as follows:
  (24) $$newDol_{i}=K_{i}+\frac{Dol_{i}-K_{i}}{DK_{i}}R_{2}$$
  where the surrounding radius $R_{2}=\left(\frac{e_{d}-2}{e_{d}}\right)DK_{i},e_{d}>2$.
(2) If $DK_{i}>R_{1}\&DK_{i}\geq DKL_{i}$, $Dol_{i}$ moves in a random way to obtain a new dolphin, as below:
  (25) $$newDol_{i}=K_{i}+\frac{Random}{\left\|Random\right\|}R_{2}$$
  where the surrounding radius $R_{2}=\left(1-\frac{DK_{i}Fit(L_{i})+(DK_{i}-DKL_{i})Fit(K_{i})}{e_{d}DK_{i}Fit(L_{i})}\right)DK_{i}$.
(3) If $DK_{i}>R_{1}\&DK_{i}<DKL_{i}$, we obtain a new dolphin $newDol_{i}$ as follows:
  (26) $$newDol_{i}=K_{i}+\frac{Random}{\left\|Random\right\|}R_{2}$$
  where the surrounding radius $R_{2}=\left(1-\frac{DK_{i}Fit(L_{i})-(DKL_{i}-DK_{i})Fit(K_{i})}{e_{d}DK_{i}Fit(L_{i})}\right)DK_{i}$.

If the iterative termination condition is fulfilled, the DSA ends. Otherwise, the DSA gets into the search stage again.

The standard DSA updates the position of each dolphin and expands child dolphins to search for the optimal solution. However, it should be pointed out that a new child dolphin is fully expanded with fixed step size during the searching stage, which leads to a fall in the local optimum for one clan of a dolphin group. Moreover, the positions of the dolphins are updated based on a random way in the process of dolphin updating. Therefore, the efficiency and convergence of the DSA decrease, and the obtained solution may result in low optimization accuracy or even failure.

To address these problems, we propose an adaptive step strategy and a novel learning strategy in the optimization process to balance the convergence speed and precision of the algorithm and better exchange information between dolphins.

(1) ***Adaptive step strategy***: we offer the following adaptive step parameter $\lambda_{d}$.
  (27) $$\lambda_{d}=\lambda_{0}+\frac{w_{1}Fit_{opt}}{w_{2}\ln N_{d}+w_{3}Fit_{0}}$$
  where $\lambda_{0}$ is the minimum step. $Fit_{opt}$ and $Fit_{0}$ are the current optimal fitness and the initial fitness, respectively. $N_{d}$ denotes the current number of iterations. The factors $w_{i},i=1,2,3$ are the regulation parameters.

Then, the formula for searching the new child dolphin of the improved algorithm is as follows:

(28) $$X_{ijt}=Dol_{i}+\lambda_{d}Vd_{j}t$$

(2) ***Learning strategy:*** The searching efficiency may be enhanced if the child dolphin $X_{ijt}$ learns from the information of the dolphin $L_{i}$ since $L_{i}$ is the individual optimal solution that $Dol_{i}$ finds in a single time. Hence, the position of $X_{ijt}$ can be obtained as:
  (29) $$V{d^{\prime }}_{j}={ο}_{x1}\times Vd_{j}+{ο}_{x2}\times (L_{i}-Dol_{i})/t$$
  (30) $$X_{ijt}=Dol_{i}+\lambda_{d}V{d^{\prime }}_{j}t$$

where ${ο}_{1}$ and ${ο}_{2}$ are the impact factors.

Next, the dolphin $Dol_{i}$ will learn from the individual optimal solution $L_{i}$. The dolphin $Dol_{i}$ updates itself according to information of the dolphin $L_{i}$ as follows:

(31) $$newDol_{i}=K_{i}+{ο}_{d1}\frac{Dol_{i}-K_{i}}{DK_{i}}R_{2}+{ο}_{d2}\frac{L_{i}-K_{i}}{DK_{i}}R_{2},\;\mathrm{if}\;DK_{i}\leq R_{1}$$

(32) $$newDol_{i}=K_{i}+{ο}_{d1}\frac{Random}{\left\|Random\right\|}R_{2}+{ο}_{d2}\frac{L_{i}-K_{i}}{DK_{i}}R_{2},\;\mathrm{if}\;DK_{i}>R_{1}$$

The improved DSA related to our optimization problem (21) solution is depicted in Algorithm 1. In this way, the optimal parameters can be obtained such that the mobile robot can reach the goal with energy efficiency and localizability simultaneously.

In the end, the optimal trajectory can thus be obtained through the use of this algorithm by iteration for every segment trajectory.

**Algorithm 1:** Improved DSA**Input:** the objective function *J*
**Output:** the parameters *T_i_* and *v_i_*

$\begin{array}{l} \;\;//\;Initialization\\ \;\;\;\;1:\;\mathrm{Initialize}\;\mathrm{randomly}\;a\;\mathrm{popution}\;\mathrm{of}\;N_{dol}\;\mathrm{dolphin}\;\mathrm{swarm}\;\\ \;\;\;Dol=\{Dol_{1},\;Dol_{2},\;\cdots ,\;Dol_{N_{dol}}\}\\ \;\;\;\;2:\;\mathrm{Define}\;DD_{i,j},DK_{i},DKL_{i}\;\mathrm{as}\;\mathrm{the}\;\mathrm{distance}\;\mathrm{bewteen}\;\mathrm{two}\;\mathrm{corresponding}\;\mathrm{dolphins}\\ \;\;\;\mathrm{and}\;TS_{i,j}\;\mathrm{is}\;\mathrm{the}\;\mathrm{rest}\;\mathrm{time}\;\mathrm{for}\;\mathrm{the}\;\mathrm{sound}\;\mathrm{of}\;\mathrm{moving}\;\mathrm{from}\;Dol_{i}\;\mathrm{to}\;Dol_{j}.\;\\ \;\;\;\;3:\;\mathrm{Specify}\;\mathrm{the}\;\mathrm{related}\;\mathrm{parameters}:\;\rho_{1},\rho_{2},\lambda_{0},w_{1},w_{2},w_{3},{ο}_{x1},{ο}_{x2},{ο}_{d2},{ο}_{d2}\\ \;\;\;\;4:\;\mathrm{while}\;\mathrm{the}\;\mathrm{termination}\;\mathrm{condition}\;\mathrm{is}\;\mathrm{not}\;\mathrm{satisfied}\;\mathrm{do}\\ \;\;//\;the\;search\;stage\\ \;\;\;\;5:\;\;\;\mathrm{Search}\;\mathrm{new}\;\mathrm{child}\;\mathrm{dolphin}\;X_{ijt}=Dol_{i}+\lambda_{d}Vd_{j}t\\ \;\;\;\;6:\;\;\;\mathrm{Calculate}\;\mathrm{optimal}\;\mathrm{fitness},\;Fit_{iab}=\min_{j=1,2,\cdots ,M;t=1,2,\cdots ,T_{sm}}Fit(X_{ijt})\\ \;\;\;\;7:\;\;\;\mathrm{Individual}\;\mathrm{optimal}\;\mathrm{solution}\;L_{i}=X_{iab}\\ \;\;\;\;8:\;\;\;\mathrm{if}\;Fit(L_{i})<Fit(K_{i})\;\mathrm{then}\\ \;\;\;\;9:\;\;\;\mathrm{Neighborhood}\;\mathrm{optimal}\;\mathrm{solution}\;K_{i}=L_{i}\\ \;\;10:\;\;\;\mathrm{end}\;\mathrm{if}\\ \;\;//\;the\;call\;stage\\ \;\;11:\;\;\;\mathrm{Update}\;\mathrm{the}\;\mathrm{rest}\;\mathrm{time}\;TS_{i,j}\\ \;\;//\;the\;reception\;stage\\ \;\;12:\;\;\;\mathrm{Exchange}\;\mathrm{the}\;\mathrm{information}\;\mathrm{between}\;\mathrm{dolphins}\\ \;\;//\;the\;predation\;stage\\ \;\;13:\;\;\;\mathrm{Calculate}\;{DD}_{i,j},\;{DK}_{i}\;\text{and}\;{DKL}_{i}\;\\ \;\;14:\;\;\;\text{Determine search Radius}\;R_{2}\\ \;\;15:\;\;\;\text{if}\;{DK}_{i}\leq R_{1}\;\text{then}\\ \;\;16:\;\;\;newDol_{i}=K_{i}+o_{d1}\frac{Dol-K_{i}}{{DK}_{i}}R_{2}+o_{d2}\frac{L_{i}-K_{i}}{DK_{i}}R_{2}\\ \;\;17:\;\;\;\mathrm{else}\;\mathrm{if}\;DK_{i}>R_{1}\&DK_{i}\geq DKL_{i}\;\mathrm{then}\\ \;\;18:\;\;\;newDol_{i}=K_{i}+o_{d1}\frac{Random}{\left\|Random\right\|}R_{2}+o_{d2}\frac{L_{i}-K_{i}}{DK_{i}}R_{2}\\ \;\;19:\;\;\;\mathrm{else}\;\mathrm{if}\;DK_{i}>R_{1}\&DK_{i}<DKL_{i}\;\mathrm{then}\\ \;\;20:\;\;\;newDol_{i}=K_{i}+{ο}_{d1}\frac{Random}{\left\|Random\right\|}R_{2}+{ο}_{d2}\frac{L_{i}-K_{i}}{DK_{i}}R_{2}\\ \;\;21:\;\;\mathrm{end}\;\mathrm{if}\\ \;\;22:\;\;\mathrm{Calculates}\;newDol_{i}\;\mathrm{fitness}\\ \;\;23:\;\;\mathrm{Update}\;\mathrm{optimal}\;\mathrm{solution}\;K_{i}\\ \;\;24:\mathrm{end}\;\mathrm{while} \end{array}$


## 5. Simulation and Experiment Results

### 5.1. Experimental Setup and Experimental Identification of the Energy Model

**Figure 5:**
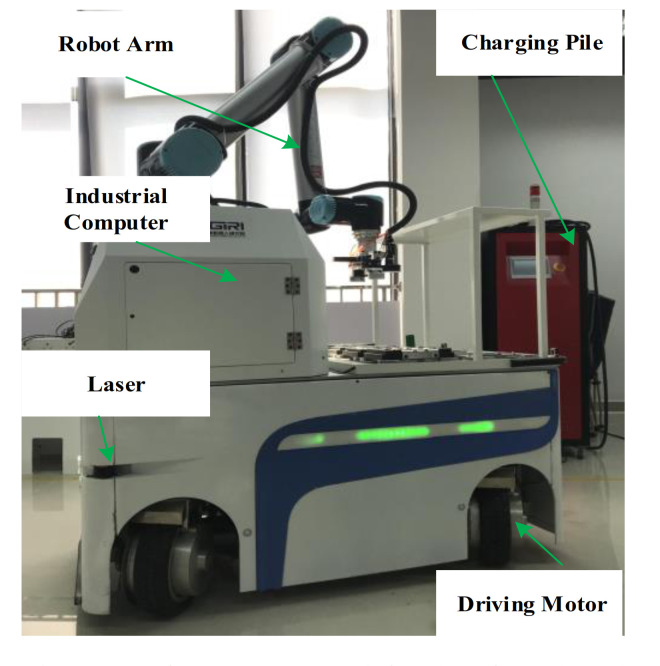
The prototype of the developed Forbot.

To test the effectiveness of the proposed optimal trajectory planning method, a four-wheel independently driven-steered mobile robot is employed in the experiments. The developed prototype robot, defined as Forbot, is shown in Figure 5. The Forbot installs two LIDARs diagonally with a 360-degree viewing angle. The Forbot has some prominent features, such as automatic charging, anti-crash measures, trackless autonomous navigation, and vision-based operating of the work-piece. The wheels with hub motors of the Forbot realize continuous wheel-ground contact so that the robot can move on an uneven floor with impurities. As each wheel of the robot has two degrees of freedom to roll and turn actively, the Forbot is able to achieve the diagonal move steer mode with a specific kinematic model [34]. The mass and inertia of the robot are 1000 kg and 60 kg·m^2^, respectively, and its battery capacity is 120 Ah. The maximum speed, maximum acceleration and minimum acceleration are 1 m/s, $0.1{\;m/s}^{2}$ and $-0.1{\;m/s}^{2}$, respectively. More specifications are shown in Table 1.

The power parameter $P_{e}$ in the energy model (1) is determined when the robot is still. In this situation, the current is stable, and we have $P_{e}=336\;w$. By performing friction coefficient experiments [35], we get μ=0.048 for the tile surface, and μ=0.086 for the carpet surface.

**Figure 6:**
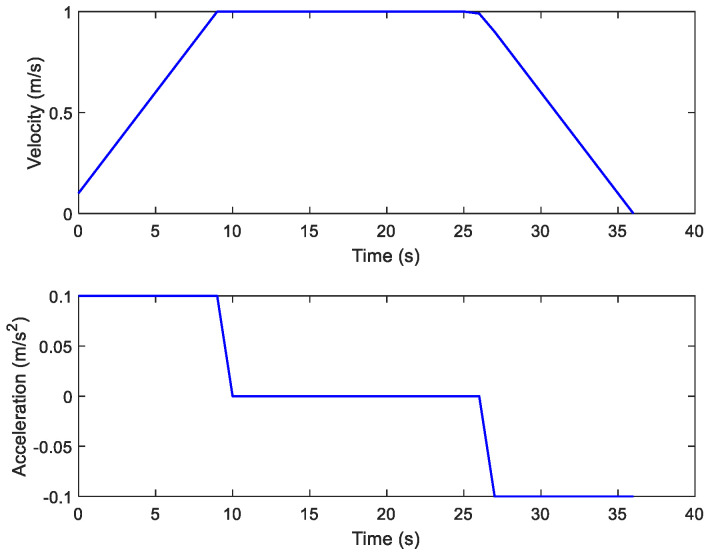
Motor’s input current with respect to the velocity.

**Figure 7:**
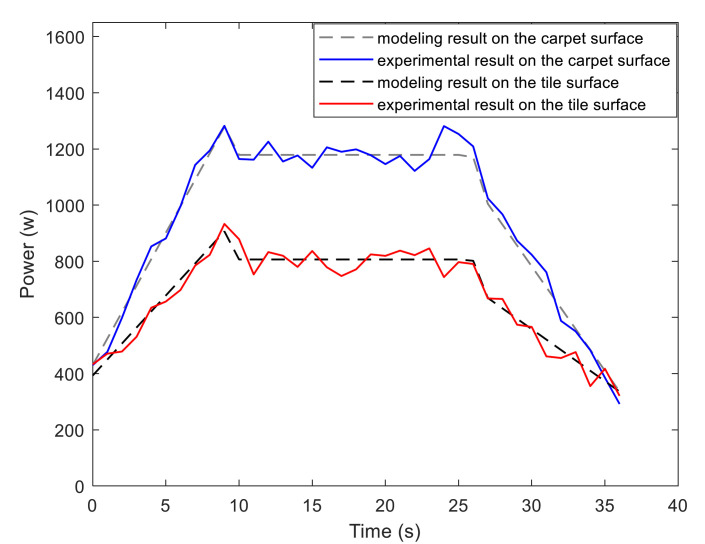
Energy model verification experiment.

To examine the validity of the energy model (1), the Forbot moves according to the designed velocity profile presented in Figure 6. We compare the experimental results and modeling results that are given by Figure 7. It can be concluded that the experimental results verify the correctness of the presented energy model.

### 5.2. Simulation Results

In this section, we first illustrate the advantages of the improved DSA for trajectory optimization. After that, the trajectory planning results in the long corridor region and circle region, with two ground surface types, are demonstrated to verify the trajectory planning performance.

(Case 1) *Improved DSA:* In this case, we will validate the advantages of the improved DSA through two experiments based on different individuals, i.e., 10 individuals and 100 individuals. Regarding the computational efficiency, we compare the classic swarm intelligence algorithms, such as the standard PSO algorithm [12], standard ABC algorithm [13], and the traditional DSA. The comparison experiments are carried out on the computer with 2.2 GHz CPU and 8 GB RAM. The initializations of dolphins are distributed randomly and evenly. The related parameters are set as $\lambda_{0}=1$, $\rho_{i=1,2}=[0.2,0.8],$ $w_{i=1,2,3}=[0.27,0.28,0.45],$ ${ο}_{di=1,2}=[0.75,0.25],$ ${ο}_{xi=1,2}=[0.75,0.25],$ which are decided through experiments to find optimal values. To ensure fairness and show consistency, the experimental results are achieved by performing each experiment 20 times independently, and the algorithm returns when the convergence condition meets the convergence accuracy or the maximum iterations.

**Figure 8:**
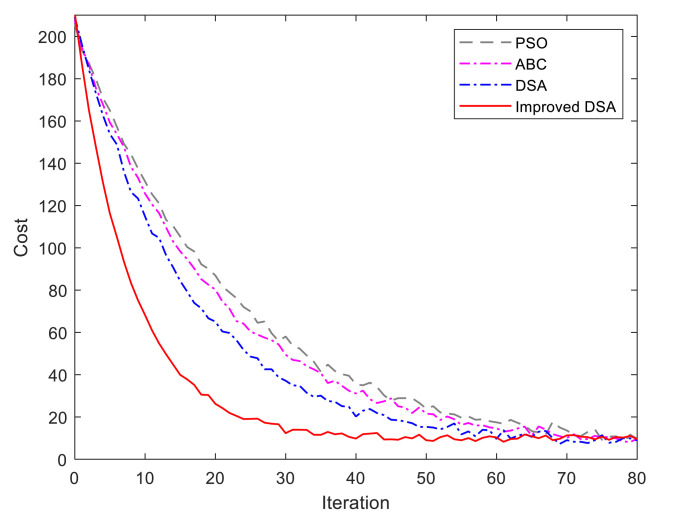
Evaluation of improved DSA algorithm under 10 individuals.

**Figure 9:**
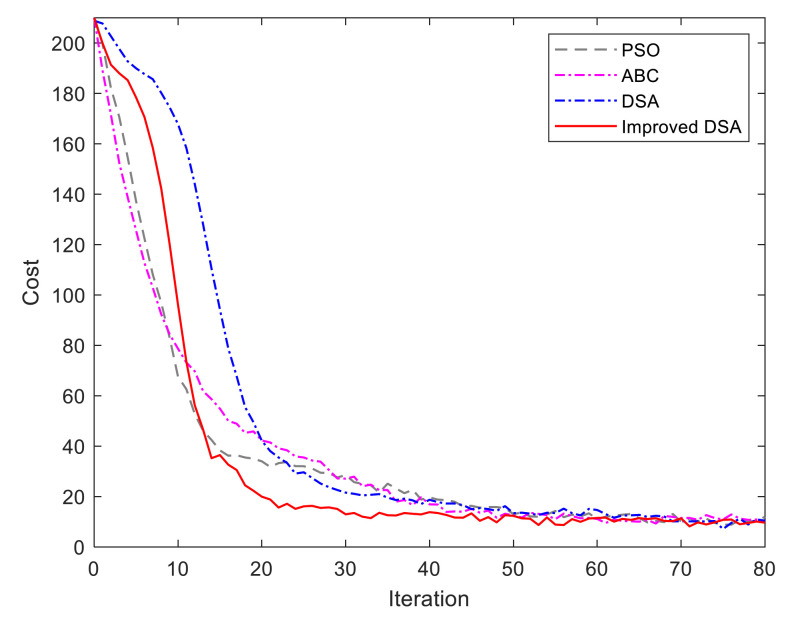
Evaluation of improved DSA algorithm under 100 individuals.

Figure 8 and Figure 9 show the running iterations based on the compared algorithms for 10 individuals and 100 individuals, respectively. As shown in Figure 8, the iteration number for trajectory optimization is prominently reduced under the improved DSA algorithm. The improved algorithm can obtain the optimal results within 29 repeated trails while the traditional one requires more than 51 iterations searching for the optimal solutions. The reason is that we present an adaptive step strategy and learning strategy in the optimization process to drive the current individual to approach the best solution. In terms of Figure 9, the ABC algorithm obtains the fastest convergence rate in the initial loops while DSA is the slowest one. Gradually, the improved DSA becomes the fastest one when the iteration reaches 13. The is because the information exchanges between dolphins result in time-delay, which is proportional to the number of dolphins and the distance between dolphins.

**Figure 10:**
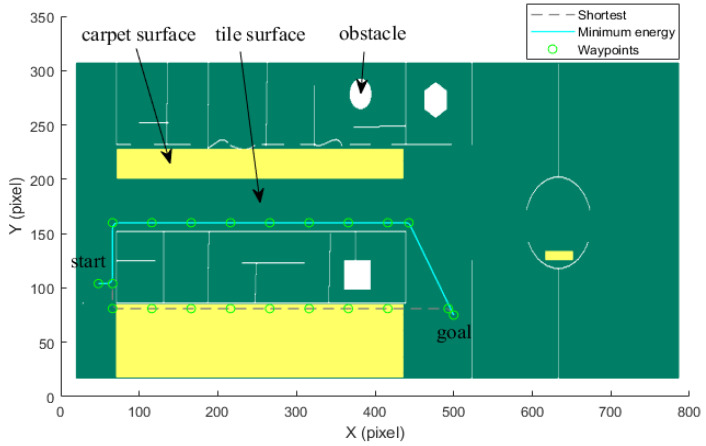
Optimal trajectory based on minimum energy in the long corridor region.

**Figure 11:**
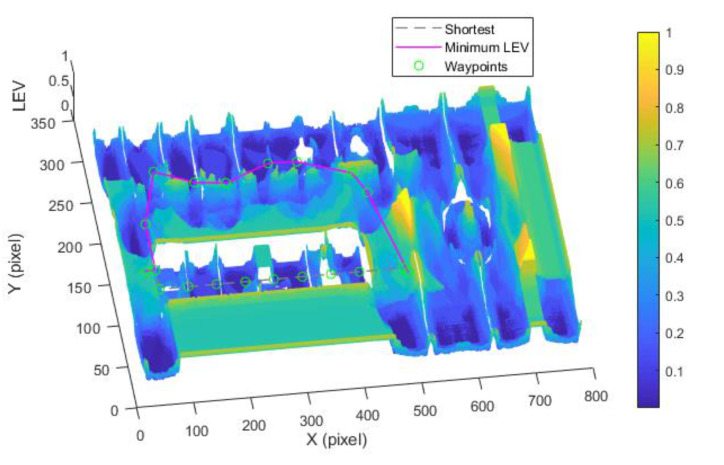
Optimal trajectory based on minimum localization error in the long corridor region.

**Figure 12:**
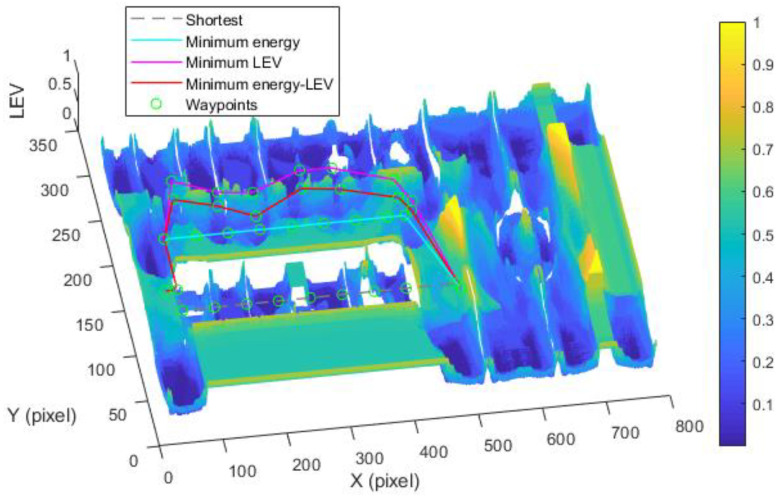
Comparison of trajectories of the case (2).

The statistics index of the computational performance is given in Table 2 and Table 3. It can be seen that our improved DSA algorithm performs better in our trajectory planning problem and has significant advantages in terms of iteration times over the other three algorithms. Moreover, the Wilcoxon’s rank-sum tests at the 5% significance level are carried out to verify the significance of the improved dolphin swarm algorithm. The Wilcoxon test results are shown in Table 4. In Table 4, “–”, “0” and “1” represent the best result, significantly different and not significantly different from the best one, respectively. The results show that our improved DSA algorithm outperform other three algorithms for the trajectory planning problem.

(Case 2) *Trajectory planning in a long corridor region:* This case considers the trajectory planning performance in a long corridor region with the two different ground surfaces shown in Figure 10, Figure 11 and Figure 12. Simulations on a four-wheel independently driven-steered mobile robot are carried out. For this simulation, the corresponding parameters are chosen as $D_{s}=1.2\;m$, $D_{m}=0.25\;m$, $\beta_{1}=0.5,$ $\beta_{2}=1.2$. To balance the weighting coefficients, $\alpha_{1}$ $\alpha_{2}$ $\beta_{1}$ and $\beta_{2}$ are determined by [0.2,0.8,0.2,0.8]. The start point and the goal point are located at [48, 104] and [500, 75], respectively.

**Figure 13:**
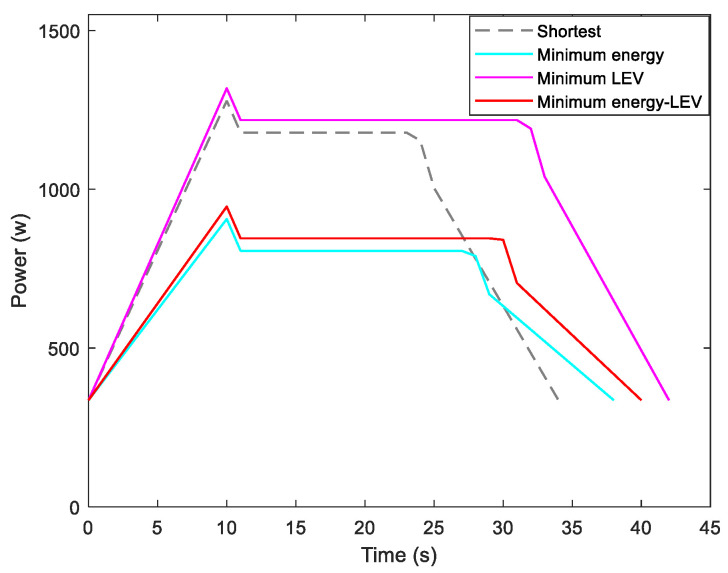
Motional power in the experiments.

**Figure 14:**
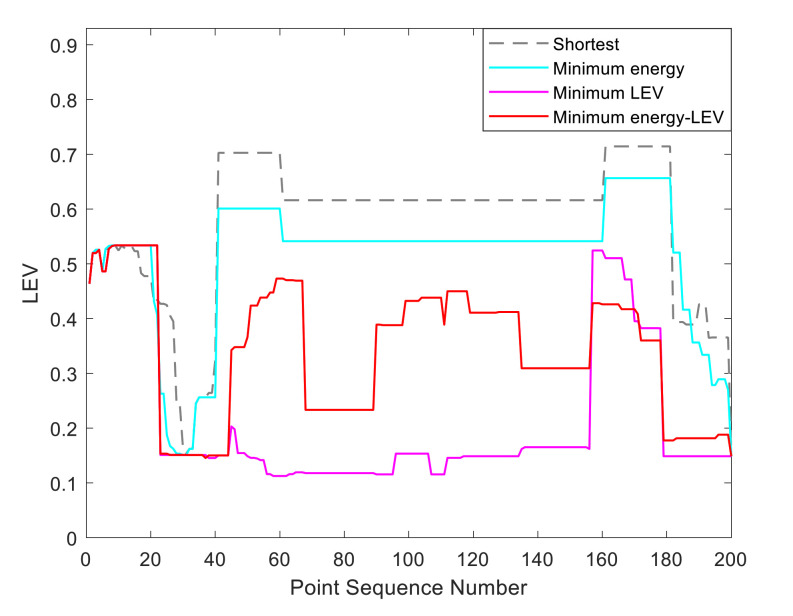
The LEV in the experiments.

By utilizing our trajectory planning method, the trajectory is generated considering localizability and energy efficiency. Figure 10 illustrates the generated trajectory only based on minimum energy consumption. As can be seen from the result, the mobile robot travels on the smooth tile surface to avoid consuming too much energy to overcome friction. Although the resulting trajectory is longer, it consumes less energy throughout the trajectory. Figure 11 shows the generated trajectory in the resulting LAM graph only based on minimum localization error. In this case, rather than moving the shortest trajectory that has the regions with high LEV, the generated trajectory is selected to be closer to featured structure regions with low LEV. As shown in Figure 12, an optimal trajectory is then generated considering localizability and energy efficiency simultaneously. Figure 13 and Figure 14 present the power and the LEV of each trajectory, respectively. Compared with other trajectories, the trajectory generated by the proposed trajectory planning method chooses the minimum total cost to balance the localization error and energy efficiency.

**Figure 15:**
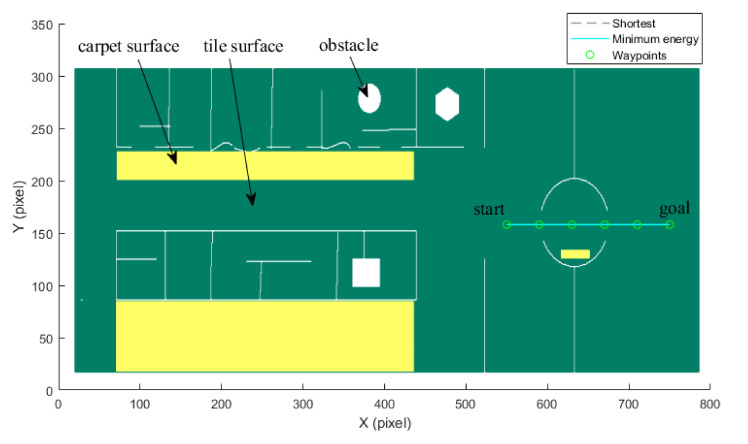
Optimal trajectory based on minimum energy in the circle region.

For quantitative analysis, energy consumption, localization error, travel distance, and total cost achieved by using the presented methods are given in Table 5. As we can see from the table, it is concluded that the proposed trajectory planning method guarantees optimal performance in a comprehensive way. To be more specific, the minimal energy-LEV trajectory has the lowest total cost of 36.99, which is enhanced by 23.65% in comparison with the shortest trajectory.

(Case 3) *Trajectory planning in a circle region:* In this case, we consider the trajectory planning performance in a circle region. As depicted in Figure 15, the experimental environment and the corresponding parameters are the same as the case (2). The difference is that the start point (550, 158) and goal point (750, 158) are located in the circle region.

**Figure 16:**
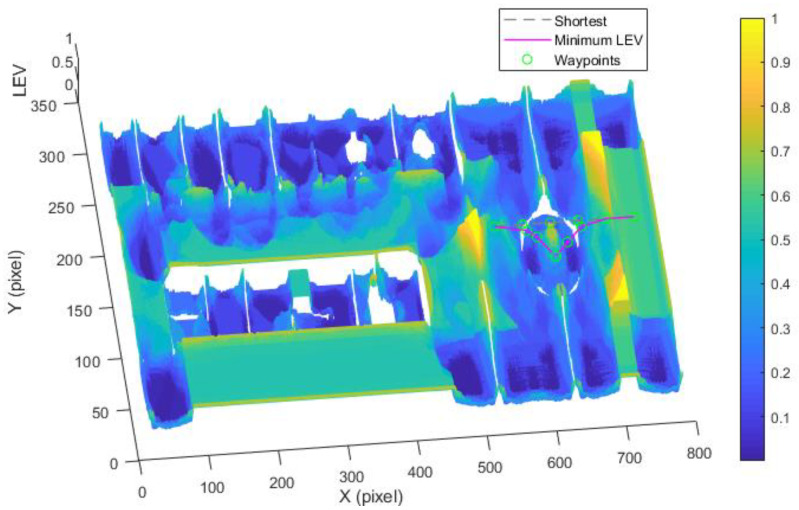
Optimal trajectory based on minimum localization error in the circle region.

**Figure 17:**
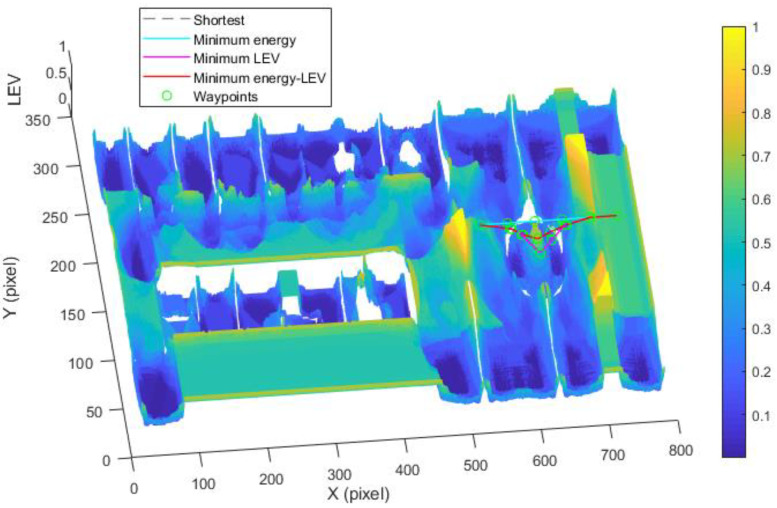
Comparison of trajectories of the case (3).

**Figure 18:**
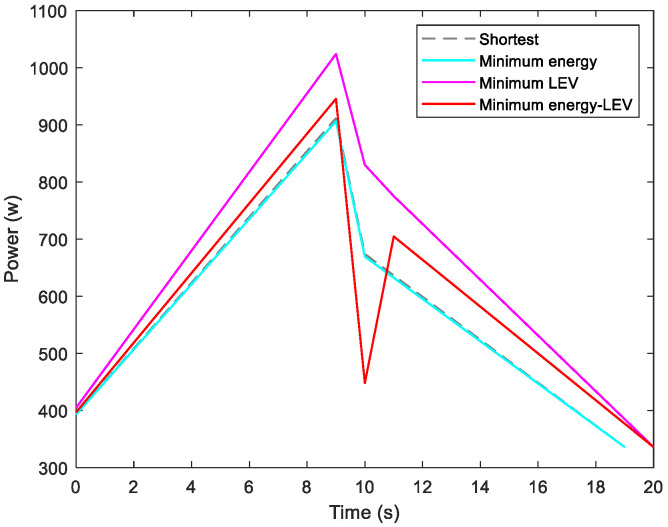
Motional power in the experiments.

**Figure 19:**
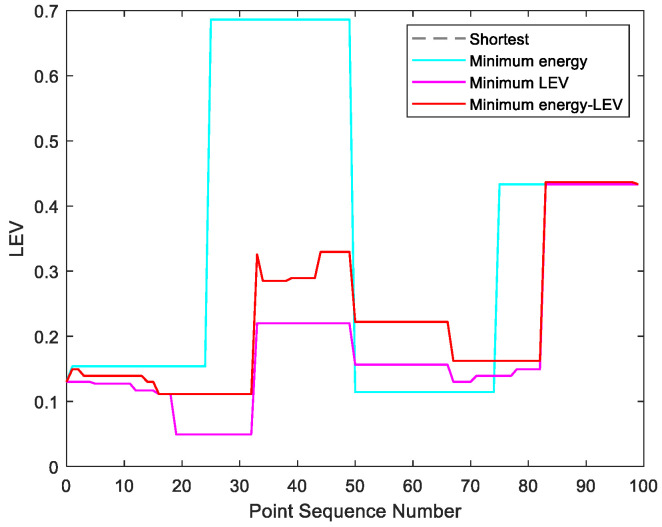
The LEV in the experiments.

Figure 15 shows the generated trajectory only considering the minimum energy consumption. This trajectory is the same as the shortest trajectory since both trajectories are on the tile surface. Figure 16 presents the generated trajectory in the resulting LAM graph only based on the minimum localization error. As we can see from the result, at the center of the circle, the LEV is very high relative to other regions nearby due to the same sensor reading regardless of the orientation. To stay away from the high LEV region, the generated trajectory is around the circumference to avoid large localization errors. In Figure 17, we can see all the resulting trajectories of this case, one of which is the optimal trajectory that is simultaneously based on energy efficiency and localizability. Figure 18 and Figure 19 show the power and the LEV of the resulting trajectories, respectively.

By examining Table 6, which presents the energy consumption, localization error, travel distance, and total cost, it can be seen that the proposed trajectory planning method achieves an optimal trajectory to maintain an acceptable level of energy consumption as well as to decrease the localization errors throughout the trajectory. Particularly, the minimal energy-LEV trajectory saves 14.93% energy consumption compared to the minimal LEV trajectory, and is 33.41% lower than the minimum energy trajectory in terms of the localization error.

### 5.3. Experiment Results

In this experiment, we verify the trajectory planning performance in an experimental environment with 799 × 985 grids, and each grid is a square with 50 mm edges. Boxes are placed into the environment, forming rich structural features, and ground surface types in the environment contain tile and carpet. The localization method proposed in [36] is applied to the Forbot robot. The sampling frequency of the control loop is specified as 10 Hz.

**Figure 20:**
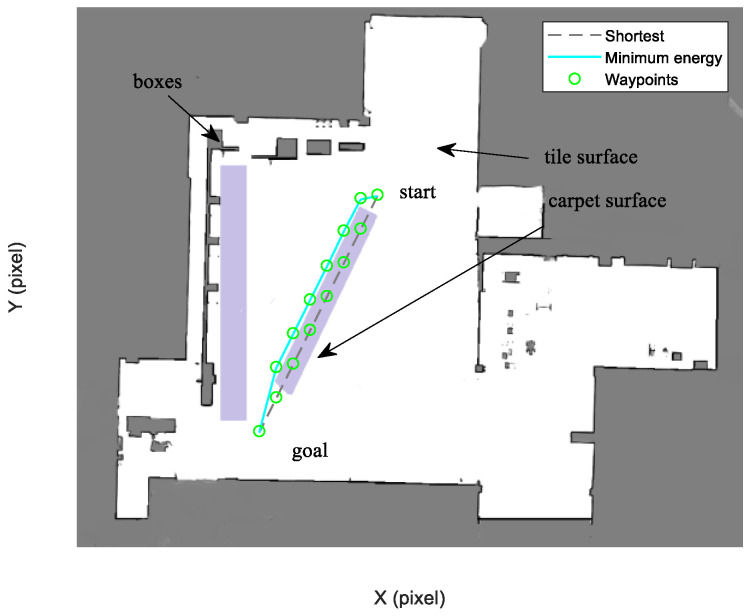
Optimal trajectory based on minimum energy.

Figure 20 shows that the Forbot approaches the goal point along the minimum energy trajectory. The dotted line represents the shortest trajectory that is mostly on the carpet surface. When the Forbot moves along the shortest trajectory, higher frictional resistance leads to more energy consumption. In contrast, although the minimum energy trajectory is longer in this case, it is found to consume less energy.

**Figure 21:**
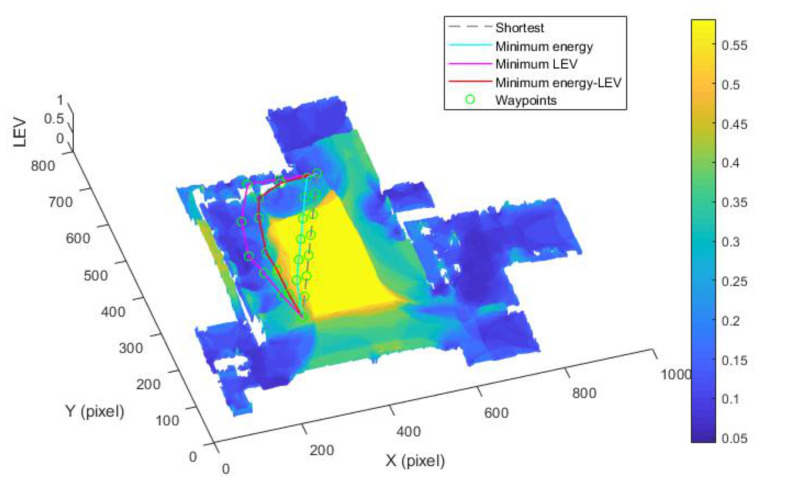
Comparison of trajectories of the case (2).

**Figure 22:**
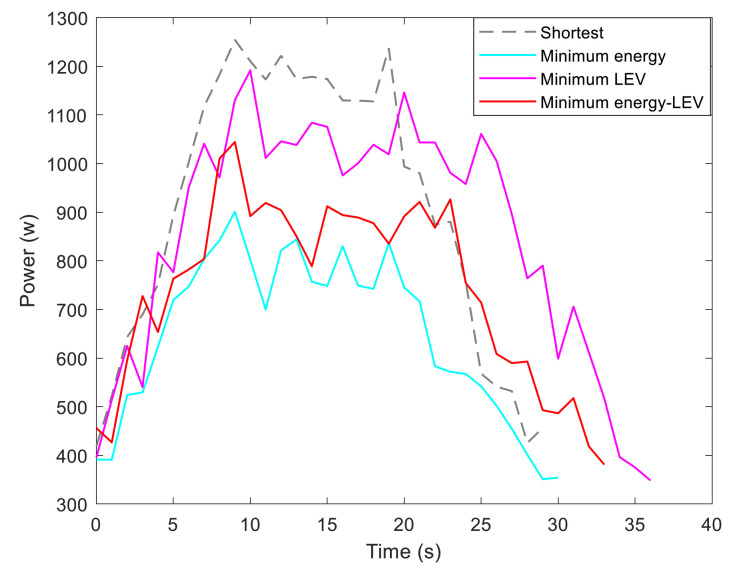
Motional power in the experiments.

**Figure 23:**
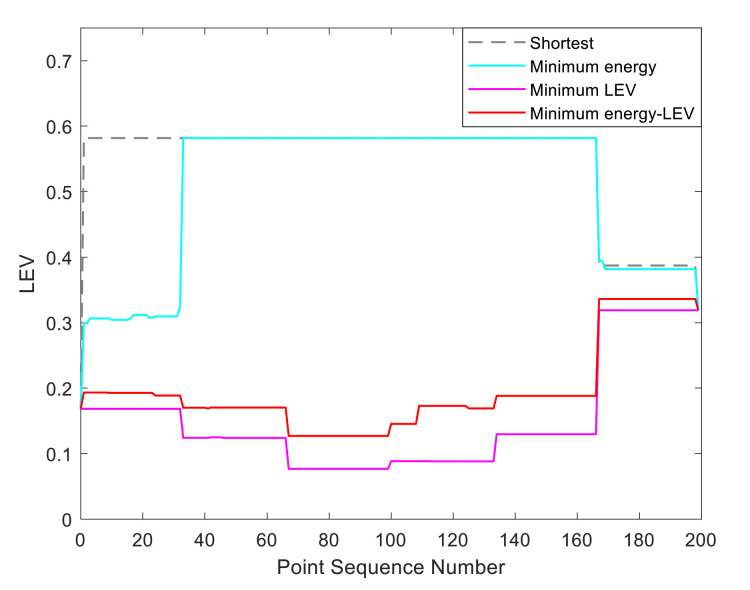
The LEV in the experiments.

As shown in Figure 21, four resulting trajectories, i.e., shortest, minimum energy, minimum LEV, and minimum energy-LEV, are generated in the LAM graph. The waypoints (green) are drawn along the trajectory in Figure 21. Figure 22 and Figure 23 present the power and the LEV profile of the experiment, respectively. As can be seen intuitively, instead of taking the shortest trajectory, the minimum LEV trajectory is much closer to featured structure regions where the localization error is relatively low. The minimum energy-LEV trajectory has a tendency to balance the energy consumption and localization error as the robot moves toward the goal.

To make the comparison more clearly and directly, we provide the energy consumption, localization error, travel distance, and total cost of the experiment in Table 7. As can be seen from the table, the proposed trajectory planning method obtains a comprehensively-optimized trajectory with guaranteed minimum total cost. More specifically, the minimal energy-LEV trajectory consumes 19.58% less energy than the minimal LEV trajectory, and reduces the localization error by 61.03% compared to the minimal energy trajectory, and ends up 36.78% more than the shortest trajectory in terms of the total cost.

## 6. Conclusions

This paper proposed an optimal trajectory planning method to obtain a minimum total cost trajectory for wheeled mobile robots by balancing localization errors and energy efficiency. A novel localizability measure method based on the likelihood function was presented to explicitly quantify the localization ability of the robot over a prior map. Then, the localizability aware map was achieved such that the localization error at a given position can be effectively estimated. By incorporating an improved DSA into the trajectory optimization process, the optimal trajectory was generated during the robot motions. We carried out the comprehensive simulations and experiments. Specifically, in the real experiment, the minimal energy-LEV trajectory consumes 19.58% less energy than the minimal LEV trajectory, and reduces the localization error by 61.03% compared to the minimal energy trajectory. The results demonstrated the efficiency of the proposed trajectory planning method in localizability and energy efficiency.

The following directions will be considered in our future works: (1) integrate the neural network optimization to enhance the quality of the generated trajectory; (2) extend our trajectory planning method to multi-robot applications.

## Figures and Tables

**Table 1:** Parameters of the Forbot.

Description	Quantity	Description	Quantity
Robot Length	0.89 m	Incremental Encoder	2500 pulses/turn
Robot Width	0.53 m	Main Frequency of PC	2.2 GHz
Wheel Diameter	0.28 m	RAM of PC	8 G

**Table 2:** Simulation results in case (1): 10 individuals.

Criteria	PSO	ABC	Standard DSA	Improved DSA
Iteration times	80	69	51	29
Mean	2.3776	3.998	0.8659	0.5231
SD	0.985	1.709	0.4022	0.1988

**Table 3:** Simulation results in case (1): 100 individuals.

Criteria	PSO	ABC	Standard DSA	Improved DSA
Iteration times	63	52	46	22
Mean	0.9524	1.2462	0.4982	0.3013
SD	0.4309	0.5758	0.2097	0.1258

**Table 4:** Results of Wilcoxon’s tests of the four algorithms.

Simulation	PSO	ABC	Standard DSA	Improved DSA
10 individuals	1	1	1	–
100 individuals	1	1	0	–

**Table 5:** Simulation results in case (2).

Trajectory	Energy (kJ)	LEV	Travel Distance (m)	Total Cost
Shortest	32.06	114.03	23.58	48.45
Minimum energy	26.36	101.83	27.37	41.45
Minimum LEV	42.75	43.91	31.33	42.98
Minimum energy-LEV	29.16	68.34	29.82	36.99

**Table 6:** Simulation results in case (3).

Trajectory	Energy (kJ)	LEV	Travel Distance (m)	Total Cost
Shortest	11.57	34.66	10.00	16.19
Minimum energy	11.53	34.66	10.00	16.16
Minimum LEV	14.53	19.04	10.84	15.43
Minimum energy-LEV	12.36	23.08	10.22	14.50

**Table 7:** Experimental results of trajectory planning.

Trajectory	Energy (kJ)	LEV	Travel Distance (m)	Total Cost
Shortest	27.83	109.46	19.57	44.16
Minimum energy	20.30	100.54	20.15	36.35
Minimum LEV	31.21	30.13	26.83	30.99
Minimum energy-LEV	25.10	39.18	23.82	27.92

## Data Availability

Not applicable.
